# The differential effects of sex hormone therapy on kidney function: insights into biological sex differences

**DOI:** 10.1172/JCI191907

**Published:** 2025-05-01

**Authors:** David Collister, Adeera Levin

**Affiliations:** 1 Department of Medicine, Division of Nephrology, University of Alberta, Edmonton, Alberta, Canada.; 2 Department of Medicine, Division of Nephrology, University of British Columbia and St. Paul’s Hospital, Vancouver, British Columbia, Canada.

## Abstract

There are known sex (i.e., biological) and gender (i.e., social) differences in the epidemiology and outcomes of chronic kidney disease. In this issue of the *JCI*, van Eeghen et al. provide a prospective multicenter observational study of transgender individuals initiating masculinizing and feminizing hormone therapy. Testosterone and estrogen with testosterone blockade had differential effects on kidney physiology including renal plasma blood flow, measured glomerular filtration rate, tubular biomarkers, and various proteins involved in inflammatory and repair pathways. The findings suggest that estrogen is renoprotective and that testosterone may be harmful to kidney function, but requires validation in larger, more diverse cohorts. The insights gained also need to be examined in the context of both endogenous and exogenous sex hormones in individuals over the life cycle.

## Sex differences in chronic kidney disease

There are known sex and gender differences in chronic kidney disease (CKD) occurrence and progression, as well as in the morbidity and mortality of cardiovascular and kidney outcomes (1). These differences, including a higher prevalence of CKD in females and women and a higher risk of kidney failure in males and men, may be due to differences in biological sex (2) (i.e., chromosomes, genes, sex hormones) and/or gender (i.e., identity, expression, roles) (3).

In the cisgender population, estrogen in females is thought to be protective against CKD, both endogenously or exogenously (i.e., as contraception or hormone replacement therapy) with potential differences in blood pressure and renin-angiotensin-aldosterone system activity (4). In cisgender males, testosterone is thought to be harmful to the kidneys, possibly related to concomitant estradiol deficiency due its partial conversion by aromatization (5). Notably, the literature dedicated to the mechanisms of endogenous and exogenous sex hormones on kidney physiology is lacking.

Evaluation of sex hormone therapy prospectively, using robust methodology, in individuals receiving feminizing or masculinizing therapy offers a unique opportunity to examine the effects of exogenous sex hormones on kidney structure and function. Previous studies that have evaluated the consequence of sex hormone on kidney function biomarkers may be confounded by changes in body composition due to sex hormone therapy, including muscle mass and adiposity, without accounting for any changes in glomerular filtration rate (GFR) (6–8). In this issue of the *JCI*, van Eeghen et al. present The Kidney Function in People Receiving Gender Affirming Hormone Therapy (KNIGHT) study to address this knowledge gap (9).

## Sex hormone therapy differentially affects kidney biomarkers

The KNIGHT study was a prospective observational cohort study conducted at two sites in Amsterdam and Colorado. The study recruited 23 individuals assigned male at birth and 21 individuals assigned female at birth, aged 17 to 40 years, with gender dysphoria who initiated masculinizing or femininizing hormone therapy prescribed according to standard local protocols. The individuals did not have hypertension, diabetes, or CKD. Study visits were conducted at baseline and at 3 months at which GFR was measured using plasma iohexol clearance and effective renal plasma flow (ERPF) using para-aminohippuric acid (PAH) clearance adjusted for body surface area. Tubular function was evaluated by urine and plasma biomarkers and molecular mechanisms were explored by plasma proteomics (SOMAscan 7K proteomic platform). The study was powered to detect a mean difference in measured GFR (mGFR) of 10 mL/min/1.73m^2^ (9).

Feminizing hormone therapy increased estradiol levels and decreased testosterone levels, resulting in a decreased mean arterial pressure (MAP) by 3 mmHg without affecting BMI or body composition (although the study was not powered to detect MAP at 3 months of follow-up). Masculinizing hormone therapy increased testosterone levels and did not change estradiol levels. While the treatment had no effect on MAP, it modified BMI and body composition (9).

During feminizing hormone treatment, mGFR increased by 3.6% from 85.0 to 87.9mL/min per 1.73m^2^ (*P* = 0.041), and ERPF increased by 9.1% from 564 to 619 mL/min per 1.73m^2^ (*P* = 0.022) with corresponding decreases in renal vascular resistance, afferent arteriole resistance, and afferent-to-efferent resistance ratio. No notable changes in mGFR, ERPF, or intrakidney hemodynamics were found with consistency across robust sensitivity analyses excluding spironolactone and adjusting for changes in body composition. Changes in mGFR, ERPF, and intrakidney hemodynamics correlated with changes in serum estradiol. Masculinizing hormone therapy did not have any effects on kidney mGFR, ERPF, or intrakidney hemodynamics (9).

**Figure 1:**
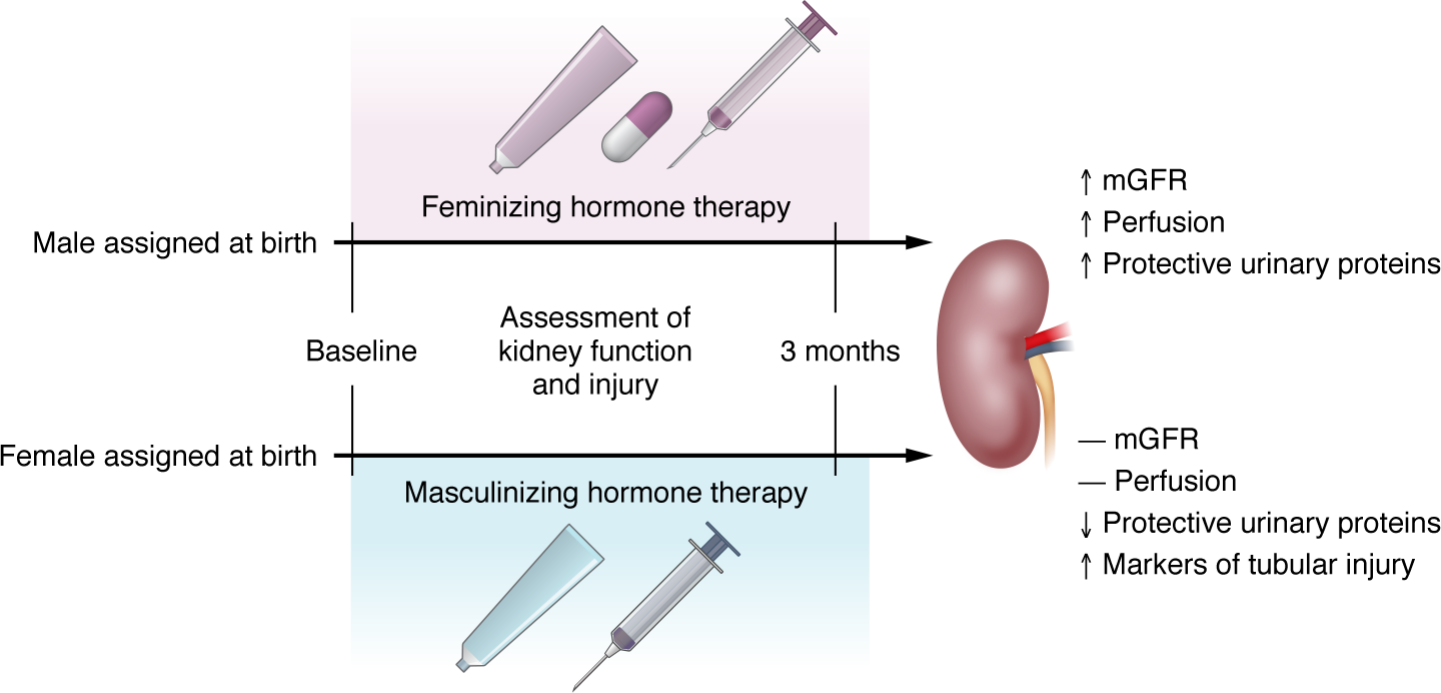
Sex hormone therapy differentially affects kidney biomarkers. The KNIGHT study evaluated kidney function and injury biomarkers in transgender individuals undergoing sex hormone treatment. Participants assigned male at birth and those assigned female at birth were assessed before and 3 months after initiating masculinizing or femininizing hormone therapy. Those undergoing feminizing hormone therapy showed increased mGFR, as determined by plasma iohexal clearance, kidney perfusion, as determined by para-aminohippuric acid clearance, and protective urinary protein markers, while individuals undergoing masculinizing hormone therapy had reductions in protective urinary proteins with increases in biomarkers for tubular injury and no changes in mGFR or perfusion. The results suggest estrogen provides renoprotection and implicates testosterone in inducing kidney damage.

Feminizing and masculinizing hormone therapy had differential effects on kidney injury biomarkers and differentially upregulated or downregulated several proteins associated with changes in mGFR and ERPF. In ingenuity pathway analyses, feminizing hormone therapy resulted in changes in 61 differentially expressed pathways including mostly downregulation, including amino acid metabolism and protein synthesis, and masculinizing hormone therapy resulted in changes in 117 differentially expressed pathways, including mostly upregulation of extracellular matrix remodeling, tissue remodeling, and immune and inflammatory responses (Figure 1) (9).

## Conclusions and future directions

van Eeghen and colleagues should be congratulated for their findings. Their use of gold-standard methods to assess mGFR and ERPF, the untargeted proteomics approach with appropriate false discovery rates, the minimal amount of missing data, and the robust sensitivity analyses (10) highlight the study’s strengths (9). Some limitations, as discussed by the authors, include the lack of inclusion of patients with CKD, short duration of follow-up, heterogeneity in hormone dose, interval, and route of administration, and the lack of consideration of gender-related factors and/or behaviors that may influence the relationship between predictors and some of the outcomes of interest (e.g., diet [ref. 11], exercise, and stressors on body weight and muscle mass).

The finding that feminizing hormone treatment is associated with increased ERPF and mGFR, without a concurrent increase in intraglomerular pressure, suggests a vasodilatory state and is consistent with our understanding of estrogen effects in female animals and cisgender women (9). Given the size of the study cohort, this study does require replication in other larger cohorts stratified by sex-hormone treatment type, dose, and route of administration, in heterogeneous populations of different ethnicity and ancestry, and with longer term follow-up (9). The reason why the administration of testosterone led to evidence of tubular injury and an upregulation of injury and repair pathways without affecting mGFR (9) requires confirmation and further (12) evaluation over longer term follow-up (13). The relative consistency of the findings across the participants in both groups (i.e., those receiving feminizing and masculinizing therapy) is encouraging (9). Additional research is needed to inform both the short-term and long-term effects of masculinizing and feminizing hormones on the various components of kidney function (i.e., GFR and tubular function) in larger diverse cohorts. If there is evidence of sustained kidney damage with exogenous testosterone, there is a need to address this both in terms of informing shared decision making and potentially testing injury prevention and mitigation strategies in those individuals. These data can also inform the issue of estimated GFR (eGFR) equation performance in transgender persons (14), to ensure appropriate identification of CKD and other clinical decision making (e.g., drug dosing and CKD dialysis planning). Longer term studies should also support evaluation of cardiovascular outcomes, automated blood pressure measurements (15), lipid changes, inflammatory and cardiac biomarkers, and cardiac imaging. Given the profound changes in GFR seen in the short term, indicating the influence of sex hormone treatment, in part, on vascular tone, it will be important to better appreciate its affects on cardiovascular functions and long-term cardiovascular risk (16).

We wonder how the results of van Eeghen et al. (9) might be applied to improved understanding of sex hormones in cisgender populations with CKD. The changes in mGFR seen in this short-term study may not seem clinically relevant at first review, but in the context of a clinical trial would be, and when contextualized over a lifetime of eGFR decline (17) could be very important. It is unlikely that estrogen will provide a therapeutic option for cisgender men given its side-effect profile or that testosterone blockers would be given to either sex to mitigate the effect of that hormone. However, appreciation of these effects may lead to strategies to mitigate these effects in those with CKD. There are also questions not answered in van Eeghen et al. (9), because of its short duration, including the following: what is the optimal magnitude and duration of change in hormone levels? How stable is kidney function with treatment over time? And how do the treatment variables affect each measures of kidney function? Larger studies would allow stronger observations between sex hormone values and changes observed and whether they are transient or sustained over time.

This study opens the possibility for many additional research questions with clinical implications (9). First, given the findings of the multiple effects of sex hormones on kidney function and processes in this transgender cohort, there is a need to better understand the impact of endogenous sex hormones over a person’s lifespan, with special attention to specific time periods including puberty, pregnancy, menopause, and andropause. In addition, for those individuals who take exogenous estrogen or testosterone, for various purposes, including contraception and hormone replacement therapy in both males and females, we have not yet explored whether there are similar changes in GFR or proteomics, as seen in this transgender population. If endogenous estrogen is found to be protective and/or endogenous testosterone is found to be harmful, then states of estrogen deficiency or testosterone excess (either primary or secondary pathology or based on genetics and population distributions) would need to have kidney outcomes considered when approaching treatment decisions. Second, it remains prudent to keep considering sex-based subgroup analyses in CKD clinical trials not only due to sex-based pharmacologic drug differences (18) (i.e., differences in pharmacokinetics including absorption, distribution, metabolism, excretion, and pharmacodynamics) but also differential sex hormone–based activation of immunologic injury and repair pathways that may be related to CKD pathophysiology or treatment targets. Lastly, the exploration of these differential pathways in kidney health and disease may identify future therapeutic targets to be validated for drug development in males, females, and intersex persons (19).

The KNIGHT study offers a myriad of insights into physiology and pathophysiology of kidney and vascular functions and leads us to the opportunity for improved understanding of sex hormones across multiple patient groups with diverse backgrounds over time and the possibility of studying the influence of exogenous and endogenous sex hormones in people with and without concomitant comorbidities (9). We will need to explore the utility of specific sex hormone levels in isolation, or in relation to each other, as well as stability of those levels over time, to capture the full complexity of their impact on cardiorenal health and disease.

## Version 1 05/01/2025

Electronic publication
